# Efficacy and safety of early prone positioning combined with HFNC or NIV in moderate to severe ARDS: a multi-center prospective cohort study

**DOI:** 10.1186/s13054-020-2738-5

**Published:** 2020-01-30

**Authors:** Lin Ding, Li Wang, Wanhong Ma, Hangyong He

**Affiliations:** 1 Department of Respiratory and Critical Care Medicine, Beijing Institute of Respiratory Medicine, Beijing Chao-Yang Hospital, Capital Medical University, No. 8 Gongren Tiyuchang Nanlu, Chaoyang District, Beijing, 100020 China; 2 Beijing Key Laboratory of Respiratory and Pulmonary Circulation Disorders, Beijing, China; 3 Beijing Engineering Research Center for Diagnosis and Treatment of Pulmonary and Critical Care, Beijing, China; 4 Department of Respiratory and Critical Care Medicine, Beijing Luhe Hospital, Capital Medical University, Beijing, China; 5 Department of Respiratory and Critical Care Medicine, Dali Bai Autounomous Prefecture People’s Hospital, Dali, China

**Keywords:** Acute respiratory distress syndrome (ARDS), Prone positioning (PP), Non-invasive ventilation (NIV), High-flow nasal cannula (HFNC)

## Abstract

**Background:**

Previous studies suggest that prone positioning (PP) can increase PaO_2_/FiO_2_ and reduce mortality in moderate to severe acute respiratory distress syndrome (ARDS). The aim of our study was to determine whether the early use of PP combined with non-invasive ventilation (NIV) or high-flow nasal cannula (HFNC) can avoid the need for intubation in moderate to severe ARDS patients.

**Methods:**

This prospective observational cohort study was performed in two teaching hospitals. Non-intubated moderate to severe ARDS patients were included and were placed in PP with NIV or with HFNC. The efficacy in improving oxygenation with four support methods—HFNC, HFNC+PP, NIV, NIV+PP—were evaluated by blood gas analysis. The primary outcome was the rate of intubation.

**Results:**

Between January 2018 and April 2019, 20 ARDS patients were enrolled. The main causes of ARDS were pneumonia due to influenza (9 cases, 45%) and other viruses (2 cases, 10%). Ten cases were moderate ARDS and 10 cases were severe. Eleven patients avoided intubation (success group), and 9 patients were intubated (failure group). All 7 patients with a PaO_2_/FiO_2_ < 100 mmHg on NIV required intubation. PaO_2_/FiO_2_ in HFNC+PP were significantly higher in the success group than in the failure group (125 ± 41 mmHg vs 119 ± 19 mmHg, *P* = 0.043). PaO_2_/FiO_2_ demonstrated an upward trend in patients with all four support strategies: HFNC < HFNC+PP ≤ NIV < NIV+PP. The average duration for PP was 2 h twice daily.

**Conclusions:**

Early application of PP with HFNC, especially in patients with moderate ARDS and baseline SpO_2_ > 95%, may help avoid intubation. The PP was well tolerated, and the efficacy on PaO_2_/FiO_2_ of the four support strategies was HFNC < HFNC+PP ≤ NIV < NIV+PP. Severe ARDS patients were not appropriate candidates for HFNC/NIV+PP.

**Trial registration:**

ChiCTR, ChiCTR1900023564. Registered 1 June 2019 (retrospectively registered)

## Introduction

Acute respiratory distress syndrome (ARDS) has a high mortality of 25~40%, even with improvement in supportive therapies. Previous studies suggest that prone positioning (PP) can increase the average ratio of arterial oxygen tension to the fraction of inspired oxygen (PaO_2_/FiO_2_) by + 35 mmHg and reduce mortality in moderate to severe ARDS, especially when combined with neuromuscular blocker (NMB) and low tidal volume ventilation, which decrease the risk of ventilator-induced lung injury (VILI) [1–5]. However, PP was only recommended in severe ARDS with PaO_2_/FiO_2_ < 100 mmHg, and the actual use of PP is less than 33% of severe ARDS patients [6].

Early use of non-invasive ventilation (NIV) can reduce the need for intubation of mild ARDS patients [7–12]. In a few observational studies [13–16], lower rates of intubation were seen among hypoxemic patients receiving high-flow nasal cannula (HFNC) than among those receiving NIV or standard oxygen therapy. HFNC provides a lower trans-pulmonary pressure (TPP) potentially resulting in less VILI than NIV.

From a theoretical and physiological point of view, NIV and HFNC may both be beneficial in patients with ARDS. However, these two techniques work via different mechanisms. NIV applies end-expiratory positive airway pressure (PEEP) and pressure support (PS). Optimally, PEEP increases functional residual capacity and opens collapsed alveoli, thereby improving ventilation–perfusion matching and reducing intrapulmonary shunt, as well as improving lung compliance, thus reducing respiratory load. The latter assists respiratory muscles during inspiration, reducing work of breathing and dyspnea. In contrast, HFNC only generates a small positive pressure spike at end-expiration that depends on the nasal airflow and the extent of mouth opening. HFNC appears to improve oxygenation primarily by flushing the nasal airspaces, reducing anatomical dead space. In addition, by delivering warm, well-humidified gas through the nostrils and avoiding the discomfort generated by the pressure that NIV masks exert on the facial skin, HFNC is tolerated much better than NIV and can be applied continuously for long periods of time [17]. The major goal of NIV and HFNC in treating ARDS is to achieve a sufficient oxygenation to avoid endotracheal intubation. However, NIV and HFNC are only “partially support” therapies. They do not address the underlying pathology of ARDS sufficiently, such as the ventilation–perfusion matching caused by atelectasis or consolidation in the dependent areas when supported with HFNC [18]. High-level PS in combination with deep inspiratory efforts could generate high tidal volumes and excessive trans-pulmonary pressures, increasing lung stress and contributing to VILI, maybe a risk for NIV failure [19]. In this regard, combining PP with these non-invasive respiratory supports in ARDS may result in better physiological effects on ventilation–perfusion mismatch, a better drainage for purulent lung infection-induced ARDS, and greater homogeneity in ARDS mechanics while receiving positive pressure support.

Based on these potential beneficial mechanisms, we performed a prospective observational study to test the hypothesis that early use of PP combined with either NIV or HFNC can avoid the need for intubation in moderate to severe ARDS patients. We also evaluated the effects of PP combined with HFNC or NIV to improve PaO_2_/FiO_2_ compared with HFNC or NIV support only, and the safety of the PP therapy in awake, non-intubated ARDS patients.

## Methods

### Study design

This prospective observational cohort study was performed in a respiratory intensive care unit (ICU) of two university teaching hospitals. This study was registered online (ChiCTR1900023564). The study was approved by the ethics committees of both participating institutions. All participating subjects or their next of kin provided written informed consent.

### Patient selection

The diagnosis of moderate to severe ARDS was made when a patient met the Berlin definition criteria [20]. ARDS patients admitted to the respiratory ICU were evaluated with arterial blood gas analysis after a PEEP of 5 cmH_2_O supported by NIV (CPAP/BiPAP mode) with FiO_2_ 0.5 for at least 30 min. ARDS patients were included if their PaO_2_/FiO_2_ was less than 200 mmHg on this level of support.

Exclusion criteria were (1) signs of respiratory fatigue (RR > 40/min, PaCO_2_ > 50 mmHg/pH < 7.30, and obvious accessory respiratory muscle use), (2) immediate need for intubation (PaO_2_/FiO_2_ < 50 mmHg, unable to protect airway or change of mental status), (3) unstable hemodynamic status, and (4) inability to collaborate with PP with agitation or refusal.

### Interventions

**Fig. 1:**
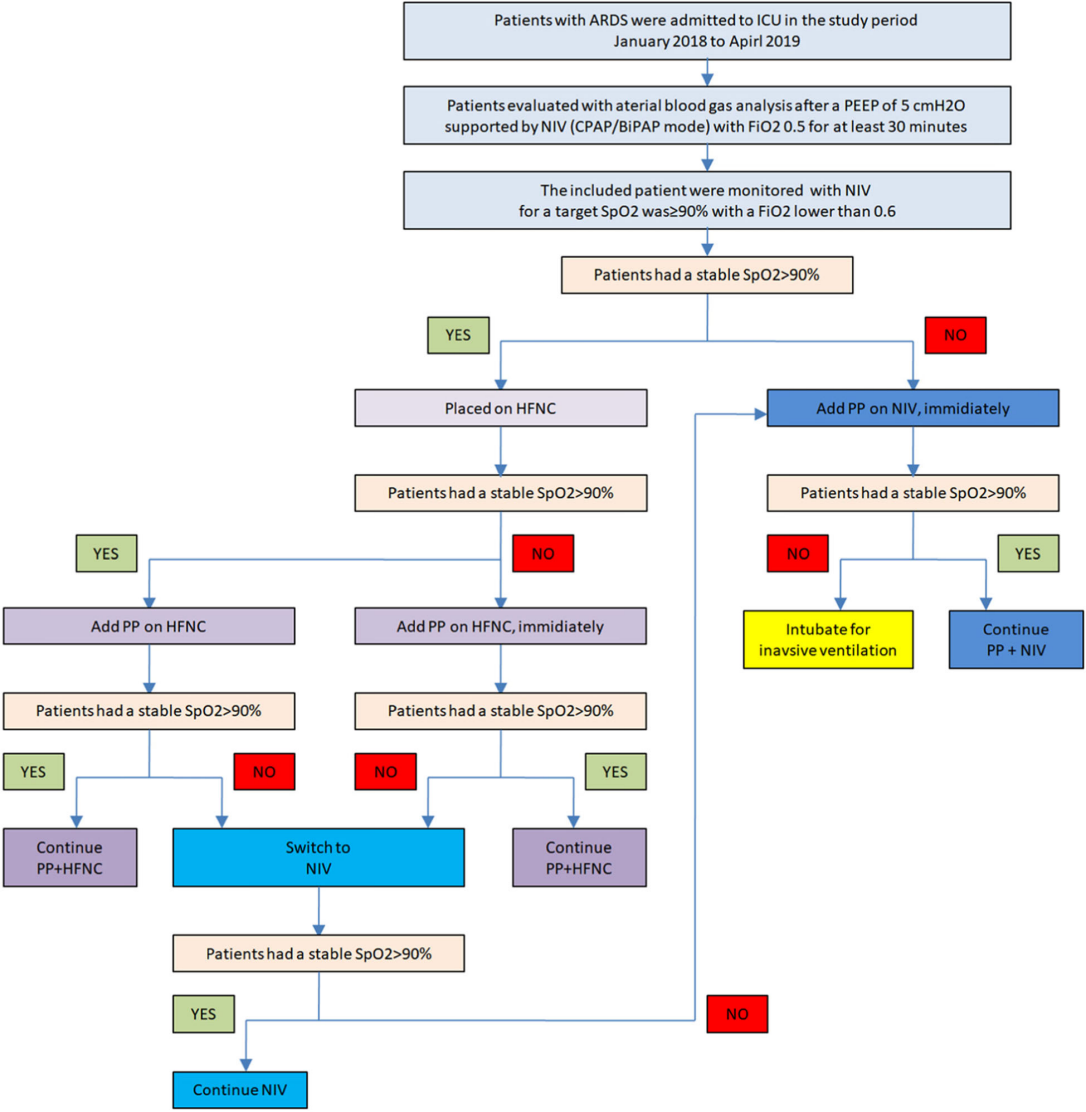
Diagram of the implementation of the interventions

Indications for PP: Included patients were all switched to HFNC after initial evaluation by NIV. The target SpO_2_ was > 90% with a FiO_2_ equal to or lower than 0.6. Patients were placed in PP in two conditions with HFNC: (1) if the patients had a stable SpO_2_, they were repositioned to PP 1 h after the HFNC was initiated; (2) if patient’s SpO_2_ was consistently < 90% on HFNC for more than 10 min, they were put in the prone position with the same setting of HFNC. If the patients had a consistent SpO_2_ < 90% when on NIV evaluation with a FiO_2_ of 0.6, they were put in a prone position with the same setting of NIV (as shown in Fig. 1). Abbreviations for four interventions applied were (1) HFNC, high-flow nasal cannula support alone; (2) HFNC+PP, high-flow nasal cannula therapy combined with prone positioning; (3) NIV, non-invasive ventilation support alone; and (4) NIV+PP, non-invasive ventilation combined with prone positioning.

Duration and frequency of PP: patients remained in PP with HFNC or NIV for at least 30 min; if patients tolerated PP well, PP would last until the patients felt too tired to maintain that position. The PP was performed at least two times a day for the first 3 days after the patient inclusion. No sedation was used during the PP. The patients were monitored by bedside respiratory therapists and nurses for their comfort and tolerance for the PP every 15 min.

Patients who received NIV were ventilated using the CPAP or bilevel positive airways pressure S/T mode (BiPAP Vision or V60; Respironics Inc., Murrysville, PA) via an oral-nasal face mask. HFNC was delivered with a max flow rate of 60 L/min and a maximum FiO_2_ of 0.9 (AIRVO2; Fisher & Paykel Healthcare Limited, Auckland, New Zealand) via a specialized nasal cannula (F&P Optiflow™).

### Outcomes

The primary outcome for the efficacy of PP combined with HFNC/NIV was the rate of avoidance for intubation. Patients who avoided intubation were classified as success while those needing intubation were classified as failure.

Secondary outcome for efficacy of PP combined with HFNC/NIV was the increase in PaO_2_/FiO_2_ from HFNC alone to HFNC+PP, to NIV alone, and to NIV+PP. The threshold of PaO_2_/FiO_2_ for successful PP cases was also assessed. Safety outcomes for PP combinations were the time duration (tolerance) for each PP therapy session.

### Data collection

The following information of all patients was collected in a data file: patients’ characteristics, including age, sex, medical history, reasons for ARDS, the laboratory and microbiology findings, and outcome and cause of death, complications including barotrauma, the different respiratory support methods used, and the time of PP. The arterial blood gases were obtained after patients received one mode of support for more than 30 min.

### Statistical analysis

Based on the 61 to 77% intubation rate for ARDS patients reported in previous studies [21, 22], we estimated a total of at least 18 subjects with an expected intubation rate of 75% in the moderate to severe ARDS patients with NIV or HFNC support would have sufficient power to detect a 40% reduction in intubation (to 45% = 75% × [1–0.4]) in the PP patients in our cases, with a confidence level [1 − *α*] = 95% and power level [1 − *β*] = 80%.

Quantitative continuous variables were given as either means (±standard deviations [SD]) or medians (with inter-quartile ranges [IQR]) were compared using the unpaired Student’s *t* test or the Mann-Whitney test. Qualitative or categorical variables were compared with the chi-square test or Fisher’s exact test. *P* values < 0.05 were considered significant. All analyses were performed with SPSS software for Windows (release 11.5).

## Results

### Patient characteristics

Between January 2018 and April 2019, a total of 20 patients were included from the two centers. The average age was 50 ± 10 years old, and 65% (13/20) were male. The main cause of ARDS was viral pneumonia, due to influenza (9 cases, 45%) and other viruses (2 cases, 10%). Ten cases met the criteria for moderate ARDS and ten cases were severe.

Twelve patients were attempted on HFNC+PP, of whom seven required escalation to NIV, with two of those patients receiving NIV+PP for further support. HFNC could not be attempted on seven patients who subsequently received NIV+PP. One patient required NIV+PP without attempting PP on HFNC when HFNC alone failed.

### Primary outcome

Eleven patients (11/20, 55%) avoided intubation, and nine patients were intubated. Three of the nine (33%) intubated patients needed extracorporeal membrane oxygenation (ECMO) support. Only one patient died in the entire group.

### Secondary outcomes

**Fig. 2:**
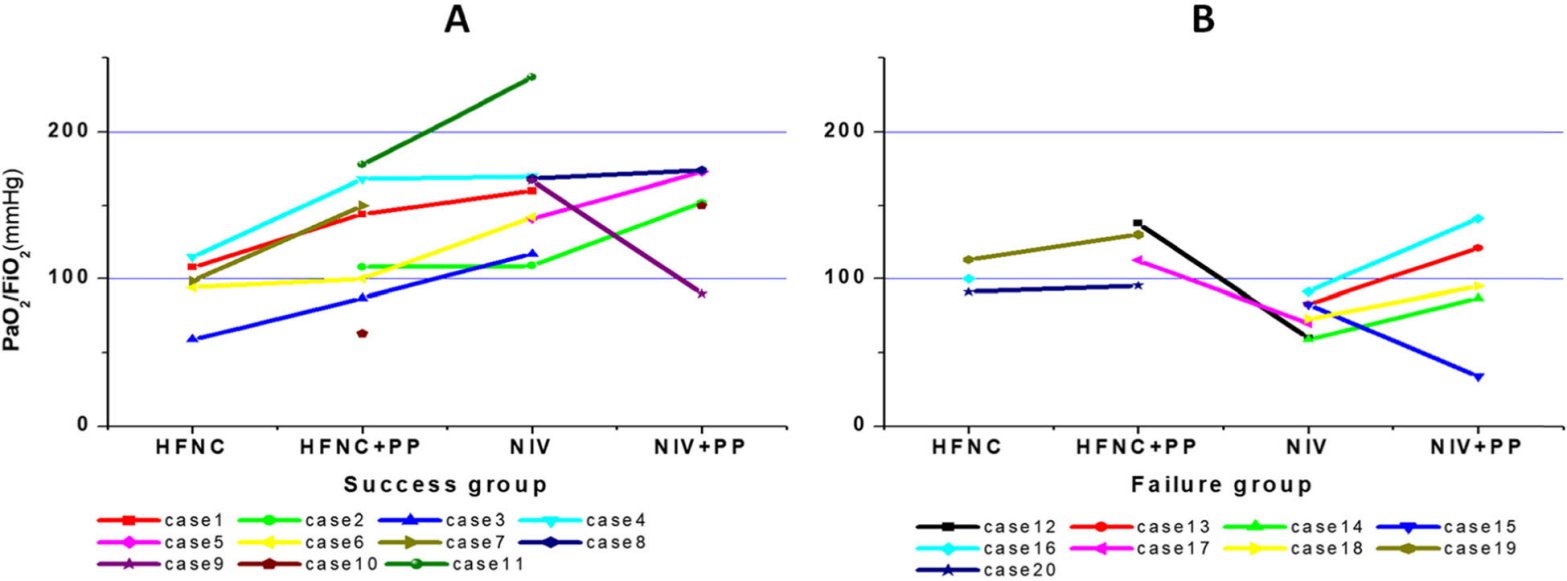
Comparison of PaO_2_/FiO_2_ of each patient with different support methods. **a** Non-intubated patients in the success group. **b** Intubated patients in the failure group

As shown in Fig. 2, the PaO_2_/FiO_2_ showed a trend of increase in transitions from HFNC to HFNC+PP, to NIV, and to NIV+PP. Only two cases had a lower PaO_2_/FiO_2_ when PP was added to NIV (cases 9 and 15), and in two cases, HFNC+PP had a higher PaO_2_/FiO_2_ than NIV (cases 12 and 17).

For the 11 patients in the success group, as shown in Table 1, eight patients received HFNC+PP, of whom six patients required change to NIV, with one patient requiring the addition of PP to NIV for further support. Three patients received NIV+PP after evaluation, and one patient received HFNC+PP and NIV+PP. PaO_2_/FiO_2_ was significantly higher in HFNC+PP than in HFNC (130 ± 35 mmHg vs 95 ± 22 mmHg, *P* = 0.016). PaO_2_/FiO_2_ had an upward trend when PP was added to NIV (166 ± 12 mmHg vs 140 ± 30 mmHg, *P* = 0.133, panel a of Fig. 2).

**Table 1:** Clinical characteristics and outcomes of patients in the success group

Case No.	Gender	Age (yo)	Cause of ARDS	Baseline PaO_2_/FiO_2_ (mmHg)	SpO_2_ (%)	PaO_2_/FiO_2_ with HFNC	PaO_2_/FiO_2_ with HFNC+PP	PaO_2_/FiO_2_ with NIV	PaO_2_/FiO_2_ with NIV+PP	Intubation	ECMO	Outcome
1	Male	43	Influenza	160	95	108	144	160		No	No	Survive
2	Male	49	Pneumonia without pathogen	109	93		108	109	152	No	No	Survive
3	Male	37	Viral Pneumonia	117	95	59	87	117		No	No	Survive
4	Male	61	Pneumonia without pathogen	170	96	115	168	170		No	No	Survive
5	Male	43	Influenza	141	93			141	173	No	No	Survive
6	Male	56	Influenza	142	95	94	100	142		No	No	Survive
7	Female	54	Influenza		92	99	151			No	No	Survive
8	Male	30	Pneumonia without pathogen	168	96			168	174	No	No	Survive
9	Male	40	Pneumonia without pathogen	167	96			167	90	No	No	Survive
10	Female	54	Pneumonia without pathogen	63	95		63		150	No	No	Survive
11	Female	48	Influenza	237	96		178	237		No	No	Survive

*ARDS* acute respiratory distress syndrome, *NIV* non-invasive ventilation, *HFNC* high flow nasal cannula, *PP* prone position, *ECMO* extracorporeal membrane oxygenation

For the nine patients in the failure group (Table 2), four patients received HFNC+PP, of whom two required NIV, and five patients received NIV+PP after initial evaluation. Excluding the one patient who was unable to cooperate with PP on NIV, PaO_2_/FiO_2_ were significantly higher in NIV+PP compared to NIV (111 ± 20 mmHg vs 77 ± 14 mmHg, *P* = 0.011, panel b of Fig. 2).

**Table 2:** Clinical characteristics and outcomes of patients in the failure group

Case No.	Gender	Age (yo)	Cause of ARDS	Baseline PaO_2_/FiO_2_ (mmHg)	SpO_2_ (%)	PaO_2_/FiO_2_ with HFNC	PaO_2_/FiO_2_ with HFNC+PP	PaO_2_/FiO_2_ with NIV	PaO_2_/FiO_2_ with NIV+PP	Intubation	ECMO	Outcome
12	Male	39	Influenza	60	90		138	60		Yes	No	Survive
13	Male	64	Viral Pneumonia	83	90			83	121	Yes	Yes	Survive
14	Male	61	Influenza	59	92			59	87	Yes	No	Survive
15	Female	65	Pneumocystis Pneumonia	83	91			83	34	Yes	Yes	Survive
16	Female	56	Influenza	91	95	100		91	141	Yes	No	Survive
17	Female	53	Influenza	70	96		113	70		Yes	No	Survive
18	Male	36	Legionella pneumonia	73	96			73	95	Yes	No	Survive
19	Female	49	Pneumonia without pathogen	130	94	113	130			Yes	Yes	Death
20	Male	63	Pneumonia without pathogen	95	90	91	95			Yes	No	Survive

*ARDS* acute respiratory distress syndrome, *NIV* non-invasive ventilation, *HFNC* high flow nasal cannula, *PP* prone position, *ECMO* extracorporeal membrane oxygenation

Comparisons of PaO_2_/FiO_2_ of patients in success and failure groups with different support methods are shown in Table 3.

**Table 3:** Comparisons of PaO_2_/FiO_2_ of patients in the success and failure groups with different support methods

	PaO_2_/FiO_2_ for low level (LL) of support (mmHg)	PaO_2_/FiO_2_ for high level (HL) of support (mmHg)	*P* value
In success patients (LL vs HL)			
HFNC vs HFNC+PP	95 ± 22	130 ± 35	0.016*
HFNC+PP vs NIV	131 ± 38	156 ± 36	0.046*
NIV vs NIV+PP	166 ± 12	140 ± 30	0.133
In failure patients (LL vs HL)			
HFNC vs HFNC+PP	102 ± 15	113 ± 25	0.349
HFNC+PP vs NIV	125 ± 18	65 ± 7	0.180
NIV vs NIV+PP	111 ± 20	77 ± 14	0.011*

*NIV* non-invasive ventilation, *HFNC* high-flow nasal cannula, *PP* prone position, *LL* low level, *HL* high level

Regarding safety, two patients could not tolerate PP. As shown in panel b of Fig. 2, all seven patients with a PaO_2_/FiO_2_ < 100 mmHg when initially evaluated on NIV only were ultimately intubated.

### Comparison of success and failure patients

Characteristics of success and failure patients are shown in Table 4. SpO_2_ was significantly higher before PP in the success group than in the failure group (95% ± 1% vs 93% ± 3%, *P* = 0.006). PaO_2_/FiO_2_ in those evaluated on HFNC+PP was significantly higher in the success group than in the failure group (125 ± 41 mmHg vs 119 ± 19 mmHg, *P* = 0.043). No significant difference in total days, frequency, and duration of PP between the successful and the failure groups was demonstrated (Table 5).

**Table 4:** Comparisons between patients avoid intubation and received invasive ventilation

	Success, *n* = 11	Failure, *n* = 9	*P* value
Male (*n*, %)	8 (73%)	5 (56%)	0.435
Age	47 ± 9	54 ± 11	0.616
Diagnosis			
Influenza (*n*, %)	5 (45%)	4 (44%)	0.965
Other viral pneumonia (*n*, %)	1 (9%)	1 (11%)	0.884
Pneumonia without pathogen (*n*, %)	5 (45%)	2 (22%)	0.104
Legionella pneumonia (*n*, %)	0 (0%)	1 (11%)	0.374
Pneumocystis pneumonia (*n*, %)	0 (0%)	1 (11%)	0.269
PaO_2_/FiO_2_ before prone position	125 ± 41	119 ± 19	0.043*
SpO_2_ (%) before prone position	95 ± 1	93 ± 3	0.006*
Moderate ARDS (*n*, %)	7 (64%)	3 (33%)	0.174
Severe ARDS (*n*, %)	4 (36%)	6 (67%)	0.174
Need for ECMO support (*n*, %)	0 (0%)	3 (33%)	0.043*
NIV combined with prone positioning (*n*, %)	5 (45%)	5 (56%)	0.653
HFNC combined with prone positioning (*n*, %)	8 (73%)	4 (44%)	0.409
Mortality (*n*, %)	0 (0%)	1 (11%)	1.000

*ARDS* acute respiratory distress syndrome, *ECMO* extracorporeal membrane oxygenation, *NIV* non-invasive ventilation, *HFNC* high-flow nasal cannula

**Table 5:** Comparisons of PP duration between success group and failure group

	Success, *n* = 11	Failure, *n* = 9	*P* value
Total days	4.3 ± 3.7	1.9 ± 1.2	0.065
Frequency during a day	2.4 ± 1.5	1.6 ± 0.5	0.130
Duration of one course of PP (h)	1.8 ± 0.7	1.9 ± 1.4	0.826

*PP* prone position

## Discussion

The main strength of our study is that, for the first time, we evaluated the early use of PP combined with HFNC/NIV in non-intubated awake patients with moderate to severe ARDS. Our study revealed that early PP combined with HFNC/NIV may avoid the need for intubation in up to half of the patients with moderate to severe ARDS; when PP was added, PaO_2_/FiO_2_ increased by 25 to 35 mmHg compared with the prior HFNC or NIV; and PP was safely performed and well tolerated by the moderate ARDS patients.

ARDS was initially reported with mortality as high as 90% [23]. Although outcome has improved in recent decades, mortality of ARDS currently approximates 32% and 45% for patients with PaO_2_/FiO_2_ of 100–200 mmHg and < 100 mmHg, respectively [24]. High mortality warrants ongoing research efforts for alternative therapeutic strategies. PP during invasive mechanical ventilation for ARDS has been extensively studied and demonstrates decreased mortality and improved oxygenation and lung recruitment. As PP was recommended for patients with a threshold PaO_2_/FiO_2_ < 150 mmHg based on previous studies, we chose to test the effects of PP in non-intubated ARDS patients with a PaO_2_/FiO_2_ < 200 mmHg, which includes moderate and severe ARDS patients.

In our study, there were 11 patients avoided intubation, including 8 (73%) patients of moderate ARDS and 3 (27%) patients of severe ARDS. Nine patients were intubated, including 2 (22%) patients of moderate ARDS and 7 (78%) patients of severe ARDS. Although no differences were statistically significant due to the small sample size of our study, moderate ARDS had a higher proportion in the successful group than in the failure group (73% vs 27%, *P* = 0.174). Moreover, SpO_2_ was significantly higher at inclusion point in the success group than in the failure group. Furthermore, although HFNC+PP or NIV+PP can improve the oxygenation compared to the HFNC or NIV single support in most severe ARDS patients, the intubation rate was very high and three patients needed ECMO for further support after intubation. Therefore, our results suggest that patients who meet the criteria for severe ARDS patients are not appropriate candidates for PP therapy. The late addition of PP to NIV in severe ARDS results in high risk for delayed intubation and treatment failure. Conversely, early application of PP in patients with moderate ARDS and an initial SpO_2_ > 95% on NIV may benefit and avoid the need for invasive ventilation.

Prone positioning may decrease the need for intubation and even mortality by improving the oxygenation of ARDS [1, 25–28]. In our study, the addition of PP actually showed an improvement for oxygenation in most cases. In the success group, when compared with HFNC or NIV support alone, PaO_2_/FiO_2_ increased significantly after PP was added. In the failure group, PaO_2_/FiO_2_ increased significantly in NIV+PP than NIV support alone. PaO_2_/FiO_2_ was also higher in HFNC+PP than HFNC. As a result, the efficacy strength for PaO_2_/FiO_2_ improvement of the four support strategies was HFNC < HFNC+PP ≤ NIV < NIV+PP.

One interesting point is that in our study, the effects of HFNC+PP were compared with those of NIV for the first time, and HFNC+PP showed similar or less effects in oxygenation improvement than NIV. When a high level of positive pressure was applied by NIV in ARDS patients with spontaneous breathing, high driving pressure plus the high level of airway positive pressure delivered by NIV may lead to a greater possibility of VILI and poor outcome [13, 19]. HFNC provides a lower level of positive pressure than NIV and thus may be better in avoiding VILI in non-intubated ARDS patients with spontaneous breathing and a high TPP. Conversely, in our study, PaO_2_/FiO_2_ was higher with NIV than HFNC+PP in the success group. Our results suggest that for the patient with good cooperation with NIV, NIV results in a higher PaO_2_/FiO_2_ than HFNC+PP and may be the first choice for improving oxygenation. In patients who cannot tolerate NIV, HFNC+PP may be an effective alternative.

When PP was performed in intubated ARDS patients, high dose of sedation or even neuromuscular blockers may be needed [4, 6]. However, in the present study, our patients generally showed good tolerance for PP while awake and receiving HFNC or NIV. The average duration of one course of PP was 2 h. In our study, ARDS was mainly caused by infectious pneumonia, and airway drainage of secretions may be important for treatment. Prone positioning in awake patients promotes better drainage of the airway and, especially when combined with HFNC, may be one reason for successful avoidance of intubation in our study.

Several limitations of our study exist. First, the small sample size was prone to bias, yielding spurious findings on statistical analysis. Increasing the sample size and collecting more cases in further study may avoid this limitation. Second, not all patients were managed with all four support strategies, possibly causing misclassification bias. Finally, this two-center observational, non-randomized, uncontrolled trial cannot prove the beneficial effects of adding PP to HFNC/NIV in early ARDS. However, our study informs the design of a future multi-center prospective randomized controlled trial (RCT) of PP in non-intubated moderate ARDS patients to answer this question.

## Conclusion

Early application of PP with HFNC or NIV, especially in patients with non-intubated moderate ARDS and with SpO_2_ > 95%, may avoid intubation. PP was generally well tolerated, and the efficacy on PaO_2_/FiO_2_ of the four support strategy was HFNC < HFNC+PP ≤ NIV < NIV+PP. Severe ARDS patients were not appropriate candidates for NFNC/NIV+PP, and the risk of complications with delayed intubation may be increased. A multi-center prospective RCT is warranted in the future in non-intubated moderate ARDS patients to get a settled conclusion.

## Data Availability

The data can be available in the medical records system of Beijing Chao-Yang Hospital.

## Abbreviations

ARDS: Acute respiratory distress syndrome
ECMO: Extracorporeal membrane oxygenation
HFNC: High-flow nasal cannula
ICU: Intensive care unit
NIV: Non-invasive ventilation
NMB: Neuromuscularblocker
PP: Prone positioning
RCT: Randomized controlled trial
TPP: Trans-pulmonary pressure
VILI: Ventilator-induced lung injury
